# Metabolomics: An Emerging “Omics” Platform for Systems Biology and Its Implications for Huntington Disease Research

**DOI:** 10.3390/metabo13121203

**Published:** 2023-12-18

**Authors:** Sumeyya Akyol, Nadia Ashrafi, Ali Yilmaz, Onur Turkoglu, Stewart F. Graham

**Affiliations:** 1 NX Prenatal Inc., 4350 Brownsboro Road, Louisville KY 40207, USA; sakyol@nxprenatal.com; 2 Department of Obstetrics and Gynecology, Oakland University-William Beaumont School of Medicine, 318 Meadow Brook Road, Rochester, MI 48309, USA; nadia.ashrafi@corewellhealth.org (N.A.); ali.yilmaz@beaumont.org (A.Y.); onur.turkoglu@corewellhealth.org (O.T.); 3 Metabolomics Division, Beaumont Research Institute, 3811 W. 13 Mile Road, Royal Oak, MI 48073, USA

**Keywords:** Huntington disease, metabolomics, biomarker discovery, NMR, mass spectrometry

## Abstract

Huntington’s disease (HD) is a progressive, fatal neurodegenerative disease characterized by motor, cognitive, and psychiatric symptoms. The precise mechanisms of HD progression are poorly understood; however, it is known that there is an expansion of the trinucleotide cytosine-adenine-guanine (CAG) repeat in the Huntingtin gene. Important new strategies are of paramount importance to identify early biomarkers with predictive value for intervening in disease progression at a stage when cellular dysfunction has not progressed irreversibly. Metabolomics is the study of global metabolite profiles in a system (cell, tissue, or organism) under certain conditions and is becoming an essential tool for the systemic characterization of metabolites to provide a snapshot of the functional and pathophysiological states of an organism and support disease diagnosis and biomarker discovery. This review briefly highlights the historical progress of metabolomic methodologies, followed by a more detailed review of the use of metabolomics in HD research to enable a greater understanding of the pathogenesis, its early prediction, and finally the main technical platforms in the field of metabolomics.

## 1. Introduction

Huntington’s disease (HD) is a rare genetically inherited neurodegenerative disorder of the central nervous system characterized by unwanted choreatic movements, behavioral and psychiatric disturbances, and dementia [1] HD affects approximately 1 in 10,000 people [2]. In general, a child whose parent (either sex) has HD has a 50% chance of inheriting the disease. It is a lifelong condition for both the individual and family and progressively worsens without treatment. 

The proposed underlying mechanism of Huntington’s disease involves several key aspects such as mutant Huntington protein formation, protein aggregation, toxicity and neuronal dysfunction, impaired autophagy and proteasomal function, mitochondrial dysfunction, transcriptional dysregulation, and excitotoxicity. The expansion of trinucleotide repeats in the HTT gene is central to the pathogenesis of Huntington’s disease. As the CAG repeats expand, the length of the polyQ tract in the huntingtin protein increases. This expanded polyQ tract is a key factor in the abnormal folding and aggregation of the mutant protein. The exact molecular mechanisms by which mHTT induces toxicity and disrupts cellular functions are complex and multifaceted. The age of onset and severity of symptoms in HD are often correlated with the length of the CAG repeat expansion, with longer repeats generally leading to earlier and more severe manifestations of the disease. Understanding these mechanisms at the molecular level is crucial for the development of targeted therapeutic strategies for Huntington’s disease [3,4]. HD appears to be more prevalent in people of European, North American, and Australian ancestry compared to those of Asian background [5]. 

To date, studies have mainly focused on alleviating symptoms rather than determining how to reverse the progression [3,6]. Although the mutant gene responsible for causing HD, htt, was discovered 30 years ago, there is still no cure for the disease. Drugs currently in clinical trials mostly target symptom control [6,7]. One of these investigational modalities includes vesicular monoamine transporter type 2 (VMAT2) inhibitors that are now used to treat HD-associated chorea. A newer VMAT2 inhibitor, deutetrabenazine (AUSTEDO™), has an improved pharmacokinetic profile and was recently approved by the U.S. Food and Drug Administration (FDA). Deutetrabenazine has fewer side effects and a longer half-life than older VMAT2 inhibitors. Despite the emerging data indicating significant symptomatic improvement of HD chorea, its long-term benefits remain unknown, and large clinical trials are warranted [8]. An important limitation of VMAT2 inhibitors is their contraindication in patients with a history of depression and suicidal ideation, which are common in HD patients. 

HD is a monogenic Mendelian disorder. Therefore, the pathogenesis of the disease is molecular, not epidemiological. Family members are at higher risk of inheriting HD than the population at large. To confirm the molecular biomarkers, a careful study of the entire family, not just primary cases, is required. As in other neurodegenerative diseases, the traits in Huntington’s are complex. The mutation leads to multiple internal alterations with multiple external conditions across the lifespan. To characterize an HD biomarker accurately, it is necessary to accumulate an appropriate collection of data that will reveal the underlying mechanism of the disease. Thus, for biomedical researchers around the world, a major goal is to identify new biomarkers and revise existing biomarkers for HD. As a result, biomarker discovery will aid in early detection and help physicians monitor the progression of the disease. For the past decade, advances in emerging technologies, such as high-resolution liquid chromatography-mass spectrometry (LC-MS) and nuclear magnetic resonance (NMR) imaging, have made it possible to explore the disease-causing mechanisms of HD. 

A significant obstacle in the realm of neurodegenerative diseases is the identification of biological signatures that correlate with disease progression or react to interventions. Underscoring the significance of early detection in Huntington’s disease is paramount, as it sets the stage for timely interventions and treatments, particularly during the crucial initial stages when their efficacy is expected to be most pronounced. Within the landscape of Huntington’s disease, evident disruptions in cellular metabolic processes become apparent. Unveiling the complexities of metabolic changes linked to Huntington’s disease, metabolomics plays a key role in shaping personalized therapeutic approaches. This in-depth comprehension opens avenues for precise and individualized interventions, potentially enhancing the effectiveness of treatments and optimizing the overall care for those impacted by Huntington’s. Metabolomics emerges as a crucial field in precisely identifying distinct metabolic alterations linked to the disease [9,10,11,12,13,14,15,16,17,18].

The aim of this review is to examine and summarize the published literature on metabolomic studies in humans, animals, and various biological fluids using existing analytical techniques for HD. The study of metabolomic phenomena not only serves as a reflection of genomics but also helps us better understand the functional activity of proteins and other molecules. The field of metabolomics has been revolutionized in the past decades, so we highlight the development of metabolomics to date. Lastly, we highlight the differences between targeted and untargeted metabolomics, their challenges, and insights into the practical considerations of targeted and untargeted metabolomics.

## 2. How It All Began: Ancient and Modern History

**Figure 1:**
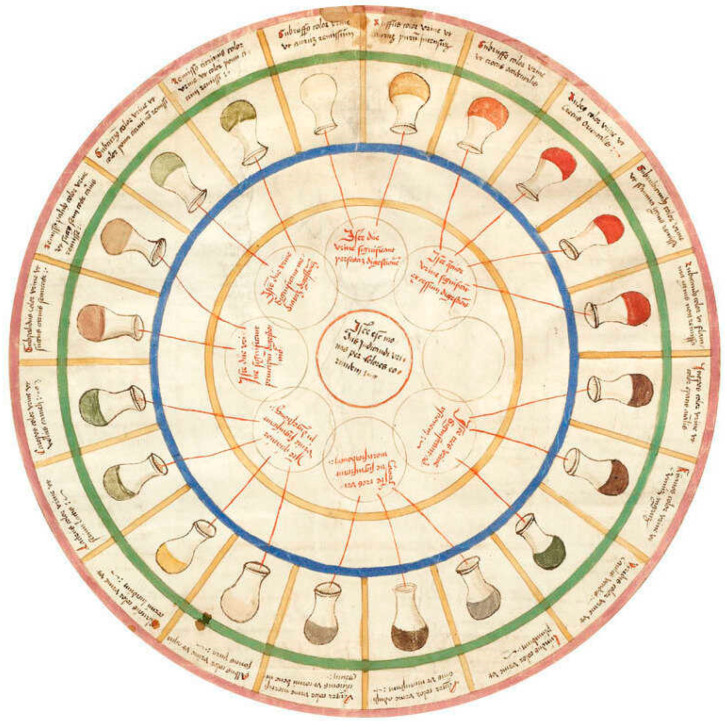
The Medieval urine wheel was used for diagnosing the diseases according to the colour, smell, and taste of the patient’s urine. The medieval wheel complements modern metabolomic research, as many diseases affect metabolism and many changes in metabolism can be detected in the urine. The urine wheel was published in 1506 by Ullrich Pindar, in his book Epiphanie Medicorum. The figure was adapted from Nicholson 2 [22].

Evidence suggests that as far back as 2600 BCE, humans have had a thirst for health-related information. Ancient Chinese cultures recognized the importance of different signs for detecting disease. For example, as early as 2000–1500 BCE, sweet-tasting urine was considered indicative of disease (now known as diabetes). Likewise, Ancient Greek history also affirms the idea that changes in tissue and biological fluids are tell-tale signs of disease [19]. Clinical testing during the Middle Ages also involved biofluid tasting (Figure 1). During the 13th century (1213–1288), metabolism was described by the polymath Ibn al-Nafis, who stated that “the body and its parts are in a continuous state of dissolution and nourishment, so they are inevitably undergoing permanent change” [20,21].

Compared with other omics, metabolomics studies change in small-molecule metabolite concentrations, which help confirm an organism’s phenotype. Metabolites are, in general, low-molecular-weight molecules (<1500 Da). The metabolome reveals important changes in the genome, transcriptome, and proteome [23]. Over the past decade, there has been a spike in interest in this field. Recent studies demonstrate that metabolomics plays a central role in areas of biology, ecology, cancer, aging, and neurodegenerative disorders [24]. In general, fields such as genomics, transcriptomics, and proteomics help us determine what might have happened or predict what may happen. In contrast, metabolomics and lipidomics offer a continuous and up-to-date picture of the state of a biological system. Of all the various omics approaches, to date, metabolomics offers the best potential for applications in biomarker discovery in systems biology [25,26]. Researchers have also shown that metabolomics holds promise for determining neuroactive small-molecule metabolites that are associated with communication between the gut microbiota and brain [27]. Great strides have been made in metabolomics applications to the field of neurology, including neural development and neurodegenerative disorders, such as Parkinson’s disease, Alzheimer’s disease, HD, and amyotrophic lateral sclerosis [28].

The naming of Huntington’s disease goes back to 1872 when George Huntington published his first and only scientific paper on chorea. Although he was not the first to describe the disease, the advantage of his study was that it documented the inheritance of the disease through two older generations (father and grandfather) of Huntington. Thus, the disease became known as Huntington’s chorea. For a long time, no significant progress was made in further understanding Huntington’s chorea [29]. Modern interest in Huntington’s chorea began in the 1980s. After researchers revealed extensive non-motor symptoms and signs, the name was changed to Huntington’s disease (HD). A study in the early 1980s confirmed a linkage on chromosome 4. It took a decade to confirm the gene for HD, and after 1993, research on HD expanded significantly [29]. With the efforts of a collaborative research group consisting of six teams in the United States and Great Britain, the htt gene was discovered.

**Figure 2:**
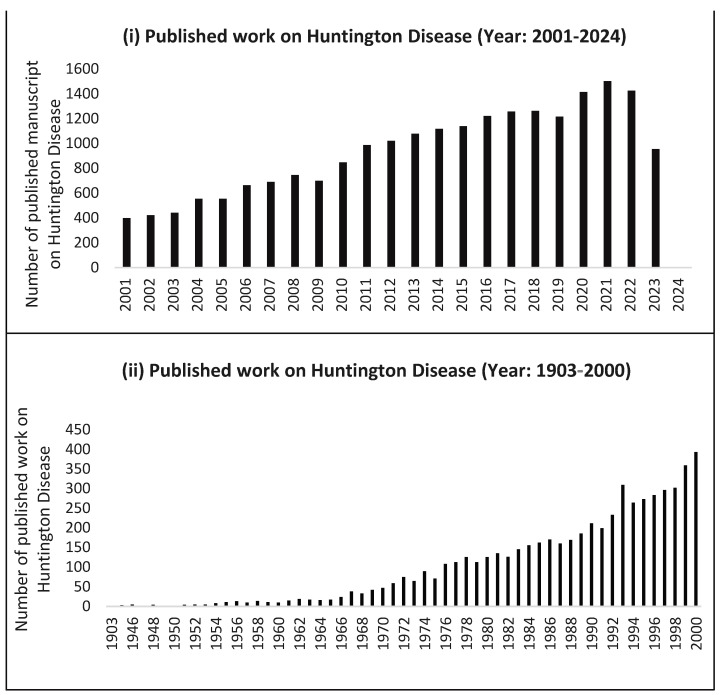
Histogram of the published manuscripts on Huntington’s disease. The chart is derived from the PubMed search engine by using the keyword “Huntington’s Disease.” The csv file format was obtained in June 2021 to plot an individual histogram of each decade. (**i**) Huntington’s research has continued to increase over the past two decades. (**ii**) The number of published manuscripts increased recently compared to early 1900 to 2000.

From the early 2000s to 2013, studies confirmed that the prevalence of HD varied around the world. The differences in the prevalence are largely due to higher cytosine-adenine-guanine [CAG] repeat sizes in normal alleles in European populations compared to Asian populations [30]. Hence, reports indicate very low rates of HD in Japan and South Africa and much higher rates in Europe (Figure 2). It is assumed that the disease spread due to the migration of affected people from northwest Europe [31,32,33].

Previously unrecognized small populations with HD disease have been reclassified more recently. These include HD cases from Lake Maracaibo, Venezuela, which are thought to derive from a single ancestor [34]. This Venezuela project has become a landmark in the history of HD because it confirmed the mapping and isolation of the HD gene. Although the discovery of the HD gene has opened new research lines and led to new models to predict the disease, the mechanism of how htt acquires abnormal functioning and cell death remains elusive (Figure 2).

Despite ongoing efforts to find a possible cure for the disease, most drugs provide only palliative relief [7]. One advantage of studying a monogenic Mendelian neurodegenerative disorder is that pre-symptomatic biomarkers will help confirm the progression of the disease. Thus, it will be valuable for clinicians to address the disease state with a quantifiable biomarker rather than a behavioral phenotype.

The history of the omics fields, including genomics, transcriptomics, proteomics, metabolomics, and lipidomics, began with the Human Genome Project, which took place from 1990 to 2003 [35]. After completing the Human Genome Project, researchers around the world began to apply its methods to explore additional biological niches, such as the transcriptome, proteome, and metabolome. Thus, omics emerged as an interdisciplinary field consisting of genomics, transcriptomics, epigenomics, proteomics, metabolomics, and lipidomics. It provides insights into the profiling and pathological complexity of systems biology. For example, genomics is used to confirm the analysis of nucleotide sequences, genome structure, and its composition. It also reveals genetic variants that can be used to confirm the disease state and the response to treatment. Genome-wide association studies (catalog: https://www.ebi.ac.uk/gwas/home, accessed on 21 July 2021) identify thousands of genetic variants associated with complex diseases in human populations [1,36]. Ribonucleic acid (RNA) is considered the primary functional readout of deoxyribonucleic acid (DNA). RNA performs an important function known as transcription and plays a vital role in translation, which is studied by proteomics. The ENCODE Consortium confirmed an integrated encyclopedia of DNA elements in the human genome. The ENCODE project confirmed that only ~3% of the genome encodes proteins and that up to 80% of the genome is transcribed (Consortium, EP 2012) [35].

In the last decade, the study of metabolomics and lipidomics has become more widespread. The history of metabolomics profiling goes back to the early 1970s. Zlatkis and colleagues performed urine profiling of 140 subjects or more [37]. Two years later, to improve the diagnosis of human disease, Thompson and Markey compared separation methods to profiling the urinary organic acids by GC-MS [38]. For over 40 years, researchers around the world accumulated publicly available libraries under the standardized conditions of 70-eV electron ionization energy. The most popular library is the U.S. National Institute of Standards and Technology (NIST) [39], but larger, less well-curated versions are also available (e.g., the Wiley registry [40], the open-access MassBank database [41], and the Golm repository) [42]. The NIST14 library accumulated 242,477 unique compounds, of which roughly one-third have recorded standardized retention times. enabling the use of two orthogonal parameters (mass spectral and retention index matching) to identify compounds [43].

**Figure 3:**
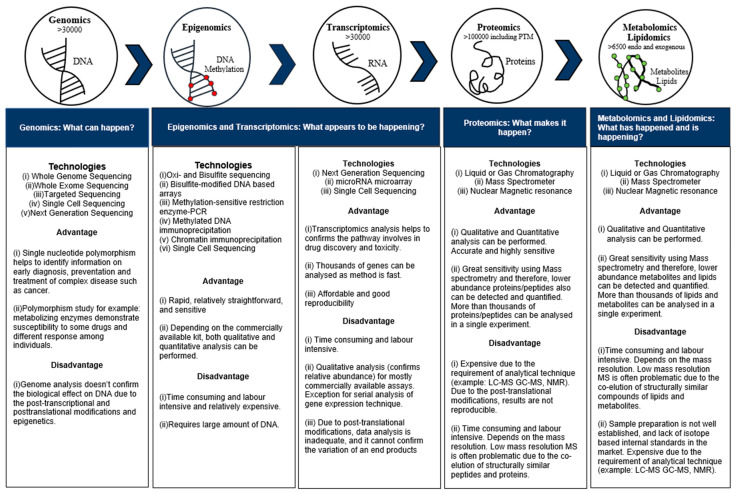
An overview of multi-omics analysis. Omics provide paramount view of biological cascade [1]. The diagram describes the advantage and disadvantage of omics analysis.

Omics is now evolving to a multi-omics approach—a new area that deserves exploration and includes the integration of genomics, transcriptomics, proteomics, and metabolomics. The advantages and disadvantages of individual omics analysis have been reviewed [1,36,44] and are summarized in Figure 3. The data integration of multi-omics analysis is complex. Thus, each laboratory needs to follow standardized multi-omics sample preparation, data acquisition, data handling, and data standardization protocols to avoid lab-to-lab variation, which can lead to biased results. The proteome contains many proteins with a wide range of physicochemical properties, such as size, charge, and hydrophobicity, which require different types of analytical techniques. Proteomics is as important as genomics and transcriptomics because it provides information about enzymes during metabolic processes. Nevertheless, proteomics studies also confirm rapid changes in cell proliferation and migration and can document not only the abundance of proteins but also dynamic protein–protein interactions [45].

## 3. Published Metabolomics Studies on Huntington’s Disease

### 3.1. Animal Studies

In early HD studies, excitotoxicity-induced cellular death and mitochondrial damage, which are two different degradation mechanisms found in the brains of HD patients, were investigated by using toxin-induced HD models—for example, quinolinic acid and 3-nitropropionate injected into mice [46]. After the htt gene was discovered, similar genetic defects were introduced into animal models. These knock-in and transgenic rodent models better represent HD progression and pathology. Emerging molecular technologies resulted in genetically modified animal models (mice and rats) in which researchers attempted to express the genetic characteristics of HD. In these animal models, mutant htt proteins were introduced by rodent germline genes. The resulting rat and mouse strains demonstrate full-length or truncated versions of a mutated htt gene, which is introduced randomly through the genomic transgene knock-in model or explicitly into the htt gene locus of rodents. New transgenic mouse models such as the R6/1 (which expresses CAG repeats 114 times) and R6/2 (which expresses CAG repeats 150 times) are the most recent transgenic mouse models. The mice have severe and early anatomical and behavioral symptoms of HD and express the human htt gene, especially part of mutant exon 1. Transgenic mice named N171-82Q express the first 171 amino acids of 82 CAG repeating htt proteins [47]. Compared with the R6/2 model, the behavioral and anatomic symptoms last longer in this model. Cloning an artificial yeast vector, including very long polyglutamine sequences, into the mouse genome is one way to create an artificial yeast chromosome (YAC1) [48], and a similar process has been used to create a transgenic rat model [49] in which the rats have serious anatomic and behavioral defects and express the CAG repeat 51 times.

The life span of rats is approximately one year longer than that of mice, and their behavior is more complex, which makes the transgenic rat model a useful tool for researching long-term treatment [50]. Some specific regions of the brain can easily be encoded with genetic mutations by using viral vectors. However, because of the technical difficulties in creating genetic models, it is more effective to use nonhuman primates [46].

Several different animal models offer different advantages for studying HD. First, large animal models have a humanoid metabolism, a brain and spinal cord closer to the size of humans, and a complex immune system are likely to mimic important neuropathological features in humans. Moreover, they have a long lifespan, which is particularly important for long-term longitudinal observation. Given the similarities in pathophysiology to humans, they not only allow for studying new treatment strategies but also provide invaluable insights into HD etiology and prevention [51,52].

The recently developed high-definition sheep model (OVT73) offers this possibility [53]. These sheep have 73 full-length repeated CAG amplification units of human cDNA transgenes. However, they lack both clear behavioral changes and structural changes in the brain [54], with only subtle neuropathology [55] and few postmortem (PM) changes in the cerebellar and liver metabolism [56], indicating that the sheep, even at 5 years of age, are still in the pre-symptomatic stage of HD. An advantage of the HD sheep model is that it can be monitored long-term to document metabolic alterations in the course of development, which enables the progression of the disease to be monitored through metabolic alterations such as rate markers or disease progression [57].

In contrast, HD mouse models, such as R6/2, have been utilized successfully to measure neuroprotective elements (for example, coenzyme Q10 [coQ10] of mitochondria), particularly when used with remacemide [58]. However, in a clinical follow-up of patients with HD, the administration of coQ10 even at very high doses did not demonstrate a meaningful attenuation of HD patients’ functional decline [59,60]. Similarly, although preclinical studies of creatine conducted in R6/2 mice showed significant results [58,61], clinical experiments in patients with early stage HD failed [62]. These treatment failures require us to ask: can preclinical studies in current high-definition animal models correctly demonstrate the response in humans? According to the Unified Huntington’s Disease Rate Scale (UHDRS) [63], a clinical rating scale to assess four domains of clinical performance and capacity in HD (motor function, cognitive function, behavioral abnormalities, and functional capacity), the performance of the metabolic biomarkers used in clinical studies is poor due to variability or long magnetic resonance imaging (MRI) response time. For this reason, a two-pronged method may be required to develop more accurate biomarkers.

In recent years, researchers have developed a highly successful transgenic minipig HD model. Libechov transgenic minipig models of HD (TgHD) [64] utilized a lentiviral method to introduce a human mHTT exon1 (1548 amino acids), which has a truncated N-terminal fragment. This fragment contains 124 glutamines (CAGCAA repeat sequence) under the human htt promoter’s control. This is the first transgenic HD pig model to successfully deliver germlines. The phenotype of the TgHD model appears to be mild disease. For up to 16 months, no aggregation formed in the brain tissue, and there were no developmental or motor deficits until 40 months [64]. Measurable and reliable phenotypes will facilitate the effective preclinical testing of the model.

Metabolomics strategies based on targeted LC/MS were used to assess biochemical changes in pre-symptomatic HD sheep in order to identify potential biomarkers [57]. The metabolites include those previously associated with HD pathology (kynurenine, urea) and metabolites that have been proposed as metabolic biomarkers of HD (amino acids that have a branched chain). The current data [57] strongly support the idea that many metabolic changes occur early in HD (even before symptoms) and may contribute to the damage and progression of the disease. Another metabolic study used the HD sheep model in which samples were collected under well-controlled conditions for 24 h to identify many more dysregulated metabolites. Although a study of patients with HD did not show changes in all the metabolites identified in the sheep model, all metabolic families or metabolites identified in the sheep had been previously recognized as being associated with HD [57]. In another previously conducted study investigating metabolite profiles using LC/MS in a transgenic sheep model of HD (OVT73) at pre-symptomatic ages, the circadian rhythmicity of metabolites (notably, phosphatidylcholines, amino acids, urea, and threonine) was found to be dysregulated in pre-symptomatic sheep. Alterations in the metabolomic profile could be used to differentiate the HD sheep from controls at 5 years of age [54].

Metabolic profiling has also been performed in a mouse model of HD, and the results show that the levels of cholesterol synthesis precursors in the brain tissue of 6-week-old R6/1 mice [65] increased, while the levels of N-acetyl aspartic acid (NAA), alanine, and aspartic acid in the striatum of late-symptomatic R6/2 mice decreased [66]. An ^1^H-NMR-based metabolomics study utilizing serum samples from rats showing symptoms of HD revealed a marked reduction in NAA levels. In those rats, 51 units of transgenes were re-amplified and transferred by CAG, thereby suggesting the early impairment of mitochondrial function in HD [67].

A significant increase in urea cycle components, arginine and citrulline, and a significant decrease in the plasma sphingolipid level occurred in the sheep model of HD. The results of investigations [68] in two mouse models and HD carriers concur that blood levels of citrulline are elevated and associated with abnormal nitric oxide and urea cycles. Progressive neurodegeneration in HD involves a volume reduction of white and gray matter as well as myelin degradation. Reduced levels of circulating sphingolipids could be related to those findings. In addition to myelination/oligodendrocyte deficiency, the destruction and/or degradation of sphingolipid synthesis likely has a major impact on cell signaling, synaptic transmission, neurotransmitter receptors, and neural function. This finding supports the observation that several urea metabolism genes, observed in HD mouse models, may cause HD symptoms. The disorder of arginase 1 (Arg1) and argininosuccinate synthase 1 (Ass1), the main components of the urea cycle, might accompany toxic metabolite collections, thereby exacerbating HD neuropathology [69,70]. Other abnormalities in the urea cycle, such as enzyme inhibition, high citrulline blood levels, and hyperammonemia, have been found in HD patients and two mouse models of HD [71,72]. Conversely, sheep models of HD show a decrease in 10 amino acids, including valine, isoleucine, and leucine (proteinogenic branched amino acids), which have been identified as potential HD biomarkers [73]. HD sheep also have reduced levels of amino acids with a branched sidechain, and the changes are seen at least 3 years after the onset of the disease. Amino acids that have a branched sidechain can potentially be used as biomarkers of HD. Based on measurements of five classes of metabolites analyzed using a logistic prediction model, it was suggested that eight metabolic biomarkers should be used as an optimized marker panel. Using these eight metabolites (threonine, C14:1, sphingomyelin (OH) C24:1, lysophosphocholine a C17:0, phosphocholine aa C36:5, phosphocholine aa C40:4, valine, and citrulline), 80% of HD sheep can be identified, with 90% confidence, as transgenic animals. These eight reliable and relatively easy-to-measure biomarkers still need to be translated into human research.

There have been many blood metabolomic analyses in the transgenic rat model of HD. A recent NMR-based metabolomics study showed that the metabolites NAA, glutamine, succinic acid, lipids, lactate, and glucose, were elevated. Possible explanations for increased glutamine and glucose concentrations observed in HD patients have been proposed: Sibson et al. [74] found that the formation of glutamate and the consumption of glucose has a 1:1 stoichiometry. The increase in glutamine levels may indicate a decrease in glutaminase activity. Glutaminase, an enzyme found in neuronal mitochondria, is responsible for converting glutamine to glutamic acid. The interruption of the glutamate–glutamine cycle could indicate a problem with energy metabolism and mitochondrial respiration [66]. Thus, the reduction of glutaminase activity prevents the conversion of glutamine [66]. Consequently, neurons lack glutamate for neurotransmission. This may also reflect mitochondrial dysfunction. There is also evidence for decreased glucose utilization and reduced succinate concentrations. These findings could be due to an impairment of glycolysis or of the TCA cycle. One possibility would be an impairment of aconitase activity, which would then lead to an increase in citrate and the inhibition of glycolysis. A decrease in aconitase activity was recently found in HD postmortem tissue as well as in the transgenic HD mice [75]. The precise nature of the metabolic defects in both transgenic HD mice and HD patients requires further investigation. The succinic acid concentration in blood of transgenic HD rats was elevated, likely because of succinite dehydrogenase (a mitochondrial enzyme that has two different functions) inhibition [67]. First, it is oxidized to fumaric acid in the citric acid cycle by succinite dehydrogenase. Second, the mitochondrial electron transport chain II complex of the respiratory chain is connected to this enzyme [76]. Mitochondrial complex II activity also decreases in HD patients [75]. Therefore, the recently discovered concentration of succinic acid is consistent with previous results. It is known that reduced NAA concentration is a sign of neuronal dysfunction [66]. In HD [66,77] and other neurological diseases [78,79], the reduction of NAA content has been well documented. In addition, in HD, the number of CAG repeats and the symptom’s durability is obviously related to N-Acetyl-L-aspartic acid (NAA) [80]. L-N-acetylaspartyl transferase, the enzyme responsible for NAA synthesis, is found only in mitochondria [79]. Because the concentration of NAA seems to be reduced by mitochondrial respiratory chain inhibitors [77], the diminished NAA content could indicate that energy production in mitochondria is impaired [66].

A few studies investigated alterations in cerebrospinal fluid (CSF) in HD. As mentioned earlier, there are limitations to studying CSF biochemically. Recent investigations revealed that the level of glucose and lactate in CSF samples are correlated with the disease status. In both serum and CSF samples, elevated glucose is thought to be a pre-symptomatic metabolic biomarker of HD. Although the literature [77,81] described evidence of diabetic patients with HD, the serum glucose levels in transgenic rat models did not change with age [49]. Thus, the observed glucose difference might be generated by a disorder in another metabolic pathway. There is ample proof of impaired glucose metabolites in HD [77,82]. In addition, the theory that HD transgenic rats lack oxidative energy metabolism is supported by a difference in lactic acid levels between transgenic animals and controls. A reduction of nicotinamide adenine dinucleotide (NAD+ to NADH) is necessary to convert glucose to pyruvate during glycolysis. Pyruvate is oxidized to acetyl-CoA then enters the citric acid cycle in the aerobic respiration of cells. Reduced NAD+ produced during glycolysis reactions is oxidized through the electron transport chain of mitochondria. However, the disruption of the citric acid cycle or the electron transport chain of mitochondria might obstruct pyruvate from entering energy metabolism driven by oxidation. Because reduced NAD+ must be re-oxidized to NAD+ to stabilize the state, another method of transferring electrons might be used; that is, pyruvate being reduced to lactic acid through a reaction catalyzed by lactate dehydrogenase. Consequently, the end-product of anaerobic glycolysis (lactic acid concentration) increases, which also results in insufficient energy metabolism [83].

### 3.2. Human Studies

Central and functional changes in the brain can be used as putative biomarkers for HD. However, one should be aware that insights provided by animal studies frequently fail to translate to humans [84], and it is very difficult to achieve clear conclusions to be drawn from animals to human [85]. Therefore, it is tremendously important, when possible, to provide optimal pathology specimens. By determining specific metabolic characteristics of HD via clinical samples, we can significantly refine our knowledge about pathologic aspects of this disease. Designated profiles can be used to screen patients and to monitor pharmacovigilance and efficacy. Such knowledge will also allow for timely diagnosis because clinical evaluation scores and HD disease progression can vary greatly.

Using LC-MS, Graham et al. accurately identified and quantified 185 metabolites in the PM frontal lobe and striatum, which are the most severely affected regions, of HD patients and compared the results to healthy controls. In this study, the findings link changes in energy metabolism, the oxidation of fatty acids, and phospholipid metabolism to HD pathology and also demonstrate significant reductions in neurotransmitters. A further significant difference in the metabolic profile of two brain regions was observed, which might be explained by extensive nerve loss in the striatum. A multivariate analysis of the data acquired from the frontal lobe and striatum shows that most of the differences in HD samples pertain to the metabolite acyl-carnitine, a naturally occurring compound that transports long-chain fatty acids to the β-oxidation system of mitochondria and phospholipids. Acyl-carnitine has important functions, such as producing platelet-activating factors, regulating enzyme activity, regulating membrane fusion and antioxidant properties, and controlling apoptosis [86]. In the lower brain, acyl-carnitine concentration in HD samples is consistent with other studies that have shown impaired oxidative damage and shortage of energy metabolism, and mitochondrial succinate dehydrogenase in the brain of patients with HD. The striatum of HD brain shows remarkably diminished glutamic acid concentration, which could be due to the reduced number of neurons in this area. One of the polyamines, spermidine, is elevated in the striatum. Using a ^1^H NMR-based metabolomics approach, Graham et al., for the first-time, investigated HD in the striatum and frontal lobe of a PM human brain [87]. They reported that the major metabolites that were significantly affected (*p* < 0.05) in the frontal lobe of HD specimens were l-leucine, myo-inositol, l-phenylalanine and tyrosine. The metabolite concentrations significantly different (*p* < 0.05) in the striatum of HD specimens were: 4-aminobutyrate, aspartate, formate, l-glutamic acid, glycine, inosine, l-leucine, niacinamide, myo-inositol, l-phenylalanine, taurine, tyrosine, uracil, urea and valine. In this study, the predictive models based on the metabolite data were able to accurately discriminate between the striatum of control subjects and HD patients. It was shown that identified metabolites could be considered potential biomarkers for detecting and monitoring HD. Moreover, as NMR and MRS are based on the same working principles, it is quite likely that in vivo magnetic resonance spectroscopy methodologies could be employed as well.

HRMS, ^1^H-NMR, and LC/MS data are difficult to compare directly due to their different quantification and extraction procedures. However, the results of these three experimental methods overlap: the affected metabolites and fold changes are similar. For example, as measured by HRMS, taurine, glutamic acid, and kynurenine in the striatum are significantly reduced [88]. Elevated urea levels were found in the postmortem HD brain [89]. It was found by ^1^H-NMR that the amounts of alanine, phenylalanine, tyrosine, glutamine, and leucine in the frontal lobe were reduced [87]. All the studies mentioned above indicate that a fully quantitative and targeted method for profiling biochemical pathways in PM human brain tissues has advantages in HD investigations. Widespread application of methods for metabolite profiling can help us understand the pathophysiology of HD and could even help to diagnose diseases in the future. The panel of prospective biomarkers identified for HD brain pathology could be a potential pre-symptomatic diagnostic test available for clinicians in the future.

The collection of serum samples for metabolic analysis is easy and minimally invasive compared to collecting other study materials such as antemortem brain tissue or CSF, either for longitudinal studies or for use in preclinical and clinical settings. However, only a few studies have investigated metabolomics of HD in blood products from HD patients. Mastrokolias et al. studied disorders of the metabolic pathway as well as metabolic markers of HD status and progression in serum [90]. In that study a targeted mass spectrometry-based metabolomics approach has been carried out to measure serum metabolite levels in HD sufferers. A well-defined linear model based on UHDRS identified 10 metabolites the concentration of which is closely related to the condition and severity of HD. Eight of the ten metabolites are phosphatidylcholines, and two are amino acids (threonine and serine). Comparison of such data with data from untargeted studies (for a description, see Section 4) showed a strong positive correlation between the same metabolites. In addition, such comparability shows that the latest results are harmonious and beneficial to prospective research on comprehensive metabolic analysis using different platforms [91,92]. Those findings are consistent with the findings of a study that described a decline in the level of phosphatidylcholine in lipid extract from the precortical area of HD rats treated with the mitochondrial toxin 3-nitropropionic acid (3-NP) [93]. Phosphatidylcholine is the main phospholipid in membranes and is thought to play an important role in cellular fate and neuronal differentiation [94]. In the past, it has been shown that choline-containing foods were effective replacement therapy particularly, a potential substrate for acetylcholine synthesis in the brain [95]. Mastrokolias et al. [90] found higher levels of threonine and serine in patients with HD compared with controls. The hydroxylated amino acid serine has a crucial function in the metabolism of pyrimidine and purine because it is the initial precursor of some nonessential amino acids in the body. Some other metabolites, such as folic acid and sphingolipids, are derived from serine because it is the main donor of carbon moieties in the biosynthetic pathway. Hence, the reason for the increase in serine may be that, in HD, the mentioned amino acids are designed to produce phospholipids, and their levels decrease with the severity of the disease. In addition, the amino acid isomers of D-serine can activate N-methyl-D-aspartatic acid (NMDA) receptors and therefore can act as neuromodulators. NMDA receptors are involved in many biochemical pathways relating to development, learning, and memory. An excessive induction of NMDA receptors might be related to HD and other neurodegenerative diseases [96].

The level of threonine, an essential amino acid, also changes in HD [90]. Serine and threonine together form the only proteinogenic amino acids with altered levels in HD. Threonine can either be thiolyzed to acetyl-CoA or converted to pyruvic acid, which might be a neuroprotective agent in neurological diseases, by increasing the outflow of glutamic acid from the brain, removing H_2_O_2_, and exerting anti-inflammatory effects [97]. In a rat model of quinolinic acid–induced HD, pyruvate administration had neuroprotective effects [98]. Thus, the increased threonine level in mutation carriers might be a compensation mechanism to produce more substrates for neuroprotective molecules, such as threonine and/or pyruvate-metabolizing enzymes.

One of the novel findings in the study by Mastrokolias et al. [90] is a negative correlation between the eight groups of phosphatidylcholine metabolites and disease progression. The reduced levels of such metabolites indicate altered lipid metabolism in neurodegenerative diseases, suggesting that the use of phosphatidylcholine could be a potential treatment option [99,100]. The finding of increased amino acid content is partially consistent with earlier studies that also found elevated serine levels, but the results were not in the striatum; they were for the Brodmann area 10 in HD patients [101]. Those results contradict the results of another study that described the reduction of serine and four other amino acids in plasma samples of carriers of the HD mutation [102]. Mochel et al. found that isoleucine, leucine, and valine metabolite levels in plasma samples of HD patients were lower than those obtained before the onset of symptoms and those from the control group [73]. This longitudinal metabolic difference can even be better monitored by utilizing multiple platforms and improving the protocols for measuring metabolites. In addition, the difference can be associated with UHDRS score thresholds used to differentiate symptoms, sample sizes, and early and mild HD patient groups. Another possible reason for this difference could be the use of serum versus plasma samples. Because the concentration of metabolites in serum samples is usually high, the analytical platform used in that study has shown serum to be more sensitive than plasma [103]. Considering the phenotypic variability of HD and the different rates of progression in HTT mutation carriers, it is necessary to validate the findings and improve the research outcomes through further studies, prior to translating them into clinical trials. The overlap of metabolite concentrations between groups (HD patients, pre-symptomatic carriers, and healthy subjects) can be reduced via additional validation experiments to differentiate concentration thresholds that can be utilized to distinguish stages of HD disease progression.

In a study by Cheng [104], a panel of metabolites from carnitine, amino acid, and phosphatidylcholine species were used in a global metabolomics screening of plasma from 15 HD patients and 17 controls to distinguish the HD patient group from the control group. The quantification of 184 related metabolites (including carnitine, amino acid and phosphatidylcholine species) in 29 HD patients, 9 pre-symptomatic HD carriers and 44 controls further showed 1 up-regulated (glycine) and 9 down-regulated metabolites (taurine, serotonin, valine, isoleucine, phosphatidylcholine acyl-alkyl C36:0, phosphatidylcholine acyl-alkyl C34:0 and, lysophosphatidylcholine acyl C20:3). These metabolic profiles strongly indicate that disturbed metabolism is involved in the pathogenesis of HD and provide novel insights into the development of novel treatment strategies for HD.

Studies on a large number of patients with HD at different phases of the disease have shown that the level of branched-chain amino acids in patients with HD is significantly lower than those of controls, which is related to disease progression and weight loss [73,105]. The other amino acid that is at a remarkably low concentration in early and symptomatic patients is leucine, a well-known activator of the mammalian target of rapamycin (mTOR), a protein kinase controlling cell growth, proliferation, and survival, leading to increased autophagic proteolysis. Gruber et al. suggested that serine and asparagine could be potential biomarkers in HD plasma [102]. However, because CSF is part of the CNS, it is more suitable for studying diseases that affect the brain. More than 450 metabolites have been identified and quantified in human CSF [106], some of which have been studied in HD [107] and other neurological diseases, such as multiple sclerosis [108], Alzheimer’s disease [109], and Parkinson’s disease [110]. A recent pilot study that conducted a cross-sectional analysis of plasma and CSF metabolomic markers in HD demonstrated significantly higher plasma levels of arginine, citrulline, and glycine, with decreases in total and D-serine, cholesterol esters, diacylglycerides, triacylglycerides, phosphatidylcholines, phosphatidylethanolamines, and sphingomyelins. In CSF, on the other hand, disease progression was associated with nominally significant increases in NAD+, arginine, saturated long-chain free fatty acids, diacylglycerides, triacylglycerides, and sphingomyelins. These data indicated altered urea cycle, glycine, and serine metabolism as underlying mechanisms for the progression of HD pathology, which warrants further investigation and validation in a larger cohort [60]. In an attempt to translate CAG expansion in the HTT gene into the clinical phenotype of HD, Herman et al. [107] examined the CSF from premanifest and manifest HD patients, as well as control participants, using LC-MS. In that study, inter-group differences demonstrated that tyrosine metabolism, including tyrosine, thyroxine, L-DOPA, and dopamine, was significantly changed in manifest vs. premanifest HD. These metabolites were shown to have a moderate to substantial association with disease severity and symptoms. Thyroxine and dopamine levels were also linked to the five-year risk of onset in premanifest HD patients. The phenylalanine and purine metabolisms were also changed, albeit less so in relation to illness severity. Reduced lumichrome levels were prevalent in mutant HTT carriers, and the levels were associated with the five-year risk of disease onset in premanifest carriers. In a recent study utilizing ^1^H NMR spectroscopy, Chang et al. investigated the alternations of lipoprotein profiles in the plasma and associated levels of metabolites as biomarkers of HD [111]. It was found the levels of HDL3-FC, HDL4-CH, HDL4-ApoA1 and HDL4-FC were significantly decreased in HD patients. There is evidence to suggest that HDL contains anti-apoptotic, antioxidant, anti-thrombotic, and anti-inflammatory properties [112]. Pradhan and his colleagues have presented a systems biology approach utilizing multi-omics data and their validation by using a yeast model to further elucidate pathways involved in the pathogenesis of HD [113]. In silico metabolomic analysis of pre-symptomatic and symptomatic HD patients showed that the deregulated pathways include metabolic pathways of various amino acids, glutathione metabolism, longevity, autophagy, and mitophagy. Peripheral biomarkers are essential for monitoring therapeutic effects and disease progression and for preventing disease symptoms. For example, total htt protein was measured in the saliva of 98 patients with manifest HD, gene-positive premanifest HD, and control subjects (matched for sex and age) [114]. Other saliva assays were also conducted using standardized ELISA, including inflammation biomarkers such as C-reactive protein, cortisol, interleukin-1β, and interleukin-6. The measurement of saliva proteins, especially htt, has potential as an important non-invasive biomarker for the onset of HD symptoms and disease progression. However, no metabolic data were found in the saliva of HD patients. Nevertheless, it is worth exploring saliva to find new biomarkers in this useful and practical biological material. In that respect, the great potential of saliva for the investigation of HD through metabolomics has been greatly demonstrated by Corey-Bloom et al. [115]. Because oxidative stress is a common pathogenic process in various neurodegenerative disorders, including HD, the authors evaluated whether uric acid (UA) may be utilized as a potentially useful biomarker. Therefore, in that study, UA levels in peripheral fluids and postmortem brain tissues from HD patients were measured, and their potential correlations with the disease and clinical information were examined. In this study, the findings in plasma were consistent with previous research in other neurodegenerative disorders, where lower blood and brain levels of UA were reported in Parkinson’s disease, amyotrophic lateral sclerosis, and Alzheimer’s disease patients [116,117,118,119]. It has been shown that UA data in saliva mimicked some features of plasma and that UA levels in saliva correlated with UA levels in plasma, suggesting that at least part of the UA present in saliva is of blood origin.

## 4. Main Analytical Platforms and Metabolomics Workflow in HD Research

**Figure 4:**
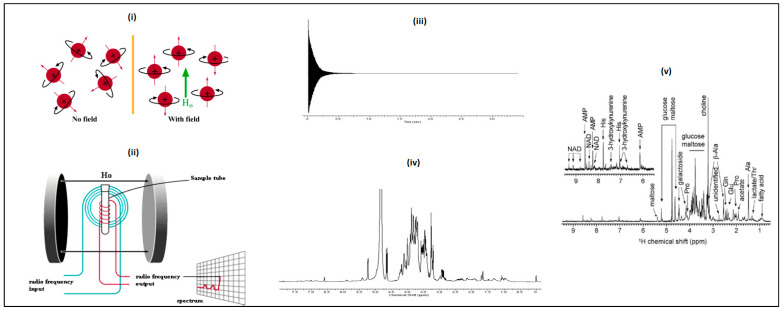
A schematic diagram of how an NMR instrument works and how spectra are produced. (**i**) Representation of nuclei without and within a magnetic field [138]. (**ii**) The spectra are produced through magnetic resonance frequency [138]. (**iii**) Initial signal received from NMR analysis prior to Fourier transformation (FT); 2-week-old plant material. (**iv**) The spectrum produced after FT. The ^1^H-NMR spectrum corresponds to the free induction decay (FID) file used to produce panel (**iii**). (**v**) ^1^H-NMR spectrum of Drosophila melanogaster metabolites [139].

Advanced analytical techniques, including nuclear magnetic resonance (NMR) spectroscopy, gas chromatography (GC), and liquid chromatography (LC) combined with mass spectrometry (MS) enable researchers to characterize past and ongoing physiological changes and to map the composition and magnitude of those changes in the metabolome. LC/MS-based metabolomics and lipidomics have been revolutionized in the past decade in the fields of chronic disease, aging, neurological disorders, biomarker discovery, microbiology, metabolic disorder, drug discovery, and precision medicine. Compared with other analytical techniques, LC/MS has the advantages of high sensitivity, selectivity, and the capability to identify known and unknown analytes of interest [120,121,122]. Nevertheless, the high reproducibility of NMR-based techniques is superior to that of MS (see Figure 4). Due to certain advantages provided by NMR and MS, to date, most metabolomic studies of HD have utilized LC/MS or NMR. Therefore, we focus on exploring the principles of those analytical platforms.

### 4.1. Nuclear Magnetic Resonance (NMR) Spectroscopy

The phenomenon of nuclear magnetic resonance (NMR) was first described in 1946 by Bloch and Purcell [123]. Atoms with an odd mass number, such as ^1^H, ^31^P, ^15^N, ^17^O, and ^13^C, have the quantum property of “spin” and behave as dipoles aligning along the axis of an applied magnetic field. During relaxation after excitation, radiofrequency signals are generated, which can be expressed as a frequency spectrum that can be further converted to chemical shift values by Fourier transformation. NMR spectroscopy is a powerful and versatile analytical technique that allows for the visualization of single atoms and molecules in various media in solution as well as in a solid state [124,125,126]. It is a nondestructive, non-invasive, non-equilibrium-disturbing technique that gives a molecular response that allows researchers to both elucidate and quantify structure simultaneously, while also providing information about the dynamics of organic molecules. With minimal sample preparation, NMR enables the fast and comprehensive detection, characterization, and quantification of various endogenous metabolites and has a high level of experimental reproducibility. Unlike most other metabolomic platforms, NMR is not restricted to biofluid or tissue extract for analysis. It is well suited for studying intact tissues, organs, and other solid or semisolid samples through solid-state NMR and magic-angle sample spinning [127,128,129,130]. Primary metabolites (compounds ubiquitous in living organisms and essential for life, such as carbohydrates, essential amino acids, and the polymers derived from them) are composed of hydrogen, carbon, nitrogen, oxygen, and phosphorus, all of which have magnetic isotopes (^1^H, ^13^C, ^15^N, ^17^O, ^31^P) that can be detected by NMR [131]. NMR spectrometers are equipped with electromagnetic radiation (EMR) sources that can be tuned to different frequencies so that they can be used to obtain NMR spectra from different kinds of nuclei. NMR spectrometers are available with field strengths up to 28 Tesla, which corresponds to a ^1^H-NMR frequency of 1.2 GHz (177). However, most metabolomic analysis is conducted on instruments that operate in the range of 300–700 MHz (Table 1) (178). NMR analysis can be undertaken using any available operating frequency; however, the higher resolution and sensitivity obtained at higher frequencies are advantageous (179), as increasing field strength increases the spectral resolution and reduces the number of overlapping signals in the spectra. Hence, the spectrometers at the upper end of the frequency range are most effective for metabolite mapping by ^1^H-NMR [132].

#### Detection Mechanism of NMR

Although NMR is an established method in many scientific fields, including chemistry, biology, physics, and medicine [135], it took almost 70 years for it to attain its current interdisciplinary status. Among spectroscopic methods, NMR spectroscopy uses the lowest irradiation energy for excitation. Consequently, the relaxation and sensitivity of NMR spectroscopy are specifically different from other spectroscopic methods [136]. Because a nucleus is a charged particle in motion, it will develop a magnetic field. ^1^H and ^13^C have nuclear spins of 1/2 and so behave similarly to a bar magnet. In the absence of a magnetic field, these nuclear spins are randomly oriented, but when a field is applied, they line up parallel to the applied field, either spin-aligned or spin-opposed [137]. The basic arrangement of an NMR spectrometer is shown in Figure 4. The sample tube is positioned in the magnetic field and excited by pulsations originating from the radio frequency input cable. The realigned magnetic fields of the nuclei induce a radio signal in the output circuit, which is used to generate the output signal. The radio signal produced by the aligned nuclei corresponds to a radio frequency in the EMR spectrum, allowing for identification [137]. Figure 4 shows the initial signals from the NMR generated in what is known as a free induction decay (FID) file. Further analysis of these signals using Fourier transformation converts the EMR frequencies into a spectrum for peak assignment (Figure 4).

Because hydrogen nuclei (protons) were the first nuclei studied by NMR, the acronym “NMR” is generally assumed to mean ^1^H-NMR. The high sensitivity of ^1^H-NMR enables any proton-containing molecule with a molecular weight of less than 30,000 Da to be detected; this high sensitivity stems from a hydrogen proton’s favorable magnetic properties and its high natural abundance (99.985%) [131]. Therefore, in practice, NMR can simultaneously detect all proton-containing compounds in a sample, including carbohydrates, amino acids, organic and fatty acids, amines, esters, ethers, and lipids, all of which are present in any tissue [140]. Simple one-dimensional (1D) ^1^H-NMR spectra have certain advantages over all other forms of NMR spectroscopy for quantifying metabolites, as data collection is carried out under fully relaxed conditions with no polarization transfer requirement [141]. Thus, ^1^H-NMR signals acquired in this way provide a real representation of the distribution of proton nuclei within the molecules and the different concentration levels of the corresponding metabolites in a complex mixture. Although NMR has the potential to provide a relatively unbiased fingerprint, typically, its output contains hundreds of overlapping peaks that render traditional NMR-based analytical practices, such as resonance assignment and peak integration, a challenge (Figure 4, panel iv) [142]. Well-isolated peaks generally scale in a discrete linear fashion, whereas overlapping peaks sum the total of the overlapping resonances. This does not affect the reproducibility of the technique but does hinder the accurate quantification of some compounds via integration. One way to address peak overlapping is to perform NMR experiments with stronger magnets and higher magnetic fields, which increases spectral dispersion. The effect of overlapping peaks can also be minimized either by using two-dimensional spectroscopy, a well-established technique in metabolomics [143], or by probing alternative NMR-active nuclei, such as ^31^P, ^15^N, or ^13^C. Whereas ^1^H-NMR spectroscopy is characterized by narrow line widths and a relatively narrow chemical shift dispersion (of ~10 ppm), ^13^C NMR spectroscopy has narrow line widths combined with a rather broad (~200 ppm) chemical shift dispersion. Therefore, ^13^C-NMR has significantly better resolution than ^1^H-NMR. However, the low natural abundance of ^13^C (~1.1%) coupled with the inherent low sensitivity of the ^13^C nucleus (compared with other common nuclei observed by NMR, such as ^1^H, ^19^F, or even ^31^P) substantially hinders the application of ^13^C-NMR spectroscopy to metabolomics, unless a signal intensity of ^13^C enhancement is achieved through ingenious spin-manipulation methods, such as distortionless enhancement by polarization transfer [144] or through the use of improved hardware and probe designs [145]. Similar to ^13^C-NMR, ^15^N-NMR can also provide characteristic spectra of wide range with narrow signals (~100 ppm), which potentially enables very accurate metabolite quantification; however, its low natural abundance (0.37%) and low gyromagnetic ratio (7.62 MHz/T) cumulatively render direct detection impossible as the ^15^N nucleus is 262,000 times less sensitive than the ^1^H nucleus. Therefore, the use of ^15^N-NMR spectroscopy in metabolomics is only possible when smart isotope tagging strategies, in which metabolites with carboxyl groups are chemically tagged with ^15^N-ethanolamine, are combined with indirect detection [146,147]. Although ^31^P is nearly 100% abundant, it has a relatively wide spectral dispersion and has a sensitivity of 6.6 × 10^−2^ relative to ^1^H. Its utility in metabolomics studies is limited because most metabolites do not contain phosphorus atoms. However, labeling lipid metabolites containing hydroxyl, aldehyde, and carboxyl groups with ^31^P reagent 2-chloro-4,4,5,5-tetramethyldioxaphospholane (CTMDP) enables the efficient use of ^31^P-NMR spectroscopy in metabolomics studies [148].

In metabolomics, two-dimensional (2D) NMR can be utilized to solve resonance overlapping problems by spreading the peaks into a second dimension by using an “orthogonal” physical property of the atom or atoms of interest, such as a covalently attached neighbor, relaxation time, or coupling constant. Consequently, 2D NMR potentially allows one to detect and identify more metabolites than is possible with 1D NMR. Indeed, different homonuclear 2D ^1^H-^1^H-NMR experiments, including many variations—total correlation spectroscopy (TOCSY) [149], correlation spectroscopy (COSY) [150,151], and nuclear Overhauser effect (NOESY) experiments [152,153,154], along with heteronuclear ^1^H,^13^C single quantum coherence (^1^H-^13^C-HSQC) [155,156] and heteronuclear multiple bond correlation (HMBC) [157] experiments—have been used routinely in metabolomics studies for many years. Diffusion-ordered spectroscopy (DOSY) [158,159] and 2D J-resolved NMR spectroscopy (J-Res) [160,161,162] are examples of other 2D NMR experiments that have also been used in several NMR-based metabolomics studies.

Homonuclear 2D NMR includes COSY and TOSCY, which provide spin-spin coupling connectivities that report which hydrogen atoms are closely located in chemical bond terms [163]. Although data collection in 2D TOCSY experiments takes a relatively long time, the 1D TOCSY is very quick (often a minute or two) and produces a relatively simple 1D NMR spectrum that is more easily analyzed.

When COSY and TOSCY are unable to provide additional information to that provided by 1D NMR, other 2D techniques are available, including HSQC [164], HMQC [165], and HMBC [164,166], all of which are indirect methods of detection. These techniques use ^1^H-NMR methods to detect, with a high degree of sensitivity and reproducibility, the presence of neighboring ^13^C and ^15^N atoms [167,168]. A greater dispersion of ^13^C (or ^15^N) chemical shifts, in comparison with ^1^H, means that more metabolites can potentially be quantified via cross-peak intensities than with conventional 1D ^1^H-NMR spectra [169,170]. One-dimensional spectroscopy combined with two-dimensional spectroscopy makes NMR a robust and reliable technique for metabolomic investigations in which high reproducibility and sensitivity are principal aims [171]. Because of its superior resolution compared with conventional 1D NMR, the potential of 2D NMR is increasingly being explored as a tool for metabolomics. However, 2D NMR requires a longer acquisition time, which makes it less suitable for high-throughput studies. Nonetheless, with the novel pulse sequences that allow for accelerated data collection, such as accelerated Double Quantum Filtered (DQF)-COSY and HSQC [150] and new probe and magnet technologies that enhance signal-to-noise ratio, NMR will likely have more prominence in the field of metabolomics.

### 4.2. Mass Spectrometry

To date, MS has become one of the most popular analytical techniques for both quantitative and qualitative metabolomic applications [172]. Over the past 55 years, MS has been transformed repeatedly, and its applications have been extended by new methods of ionization. The advent of new methods of ion generation, novel mass analyzers, and new tools for data processing have made it possible to analyze a range of substances from small organic compounds to large biological molecules. The electrospray ionization (ESI) mechanism was investigated 47 years ago by Dole and colleagues [173], but the real breakthrough occurred in 1988 when Fenn and coworkers demonstrated that ESI is suitable for larger biomolecules. The Nobel Prize in Chemistry was awarded for that work in 2002. ESI-MS allows large, nonvolatile molecules to be analyzed directly from a liquid phase and is normally coupled with a separation technique, such as high-performance liquid chromatography (HPLC) [174,175]. To date, liquid chromatography-electrospray ionization-mass spectrometry (LC-ESI-MS) is one of the most preferred analytical techniques in metabolomics.

#### 4.2.1. Mass Analyzers and the Detection Mechanism

**Figure 5:**
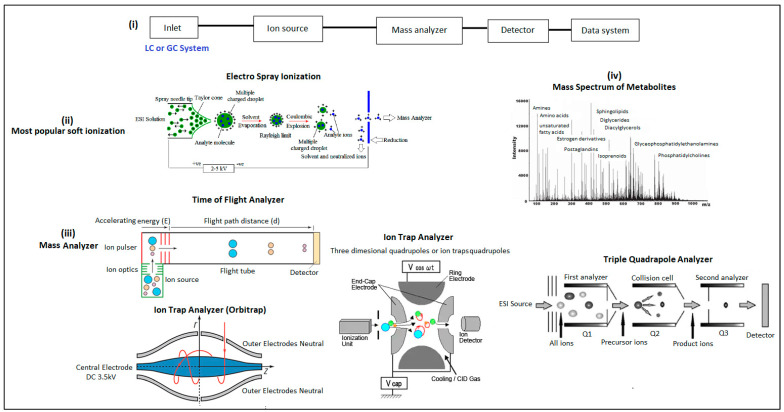
Schematic diagram of how mass spectrometry works. (**i**) Principal components of a mass spectrometer. (**ii**) How formation of ions occurs during the most used electrospray soft ionization [178]. (**iii**) Detection mechanism in time-of-fight, ion trap both quadrupole and orbitrap and triple quadrupole. (**iv**) A mass spectrum of plasma metabolites obtained by direct infusion of a sample into electrospray ionization coupled with quadrupole time-of-flight mass spectrometry [179].

The process of mass analysis in LC/MS is central to this technology and involves separating analyte ions by using a mass analyzer. Three basic types of mass analyzers are currently used in metabolomics research—ion trap, time-of-flight (TOF), and quadrupole or mass filter (Figure 5). The major advantages of quadrupole analyzers are their low cost, relatively small size, robustness, and ease of maintenance. The quadrupole mass analyzer was first described in the 1950s by the Nobel Prize-winning physicist Wolfgang Paul (not Wolfgang Pauli) [176]. A quadrupole (Q) mass analyzer has four parallel electrical rods typically with circular cross sections; two rods carry a positive charge, and the other two rods carry a negative charge. In general, a higher radio frequency (RF) is applied to two rods and the other two are linked to a direct current (DC). Ions formed in the ionization chamber are pulsed toward a quadrupole by an electrical field (~5 kV). Consequently, positively charged ions travel toward negatively charged rods, and negatively charged ions travel toward positively charged rods. Once the polarity has been changed, the ions switch their movement pathway before striking the rods and thus are transmitted through. The ions enter through a small orifice at the center of the rods and display specific trajectories based on their mass/charge number (*m*/*z*) values. Only ions with very short/narrow intervals for their *m*/*z* values have stable trajectories and successfully pass through the quadrupole rods to the detector. All other ions with lower or higher *m*/*z* values have unstable trajectories and are filtered out [177].

A single quadrupole system contains only one mass-filtering quadrupole, whereas in a triple quadrupole (QQQ) system, Q1 and Q3 act as mass filters while Q2 acts as a collisional cell. A quadrupole has limited capability in terms of mass range (usually <4000 *m*/*z*), resolving power, and the ability to perform MS/MS analysis. This disadvantage can be overcome by attaching additional quadrupoles, such as in a triple quadrupole instrument, or by linking to a quantitative time-of-flight analyzer (QToF). Time-of-flight ion separation is one of the simplest and most popular analyzers for mass spectrometers. It relies on the free flight of the ionized molecules in a drift tube before reaching the detector [173]. The time required for an ion to travel a set distance and strike a detector enables the *m*/*z* ratio to be calculated.

When an electric field is applied to a free ion, it will give the ion kinetic energy of zV, where z is the ion charge and V is the applied voltage. The flight time (t) is determined by the energy (E) to which an ion is accelerated, the distance (d) it has to travel, and its *m*/*z* ratio. There are two well-known formulae that apply to time-of-flight analysis. An example of one such formula for kinetic energy is:

E = ½mv^2^,

where E = kinetic energy, m = mass, and v = velocity. This equation shows that for a given kinetic energy, E, smaller masses will have larger velocities, and larger masses will have smaller velocities. Instead of measuring velocity, it is much easier to measure the time it takes an ion to reach the detector. The second equation is the familiar v = d/t, where v = velocity, d = distance, and t = time. This equation describes the basic time-of-flight relationship. For a given energy (E) and distance (d), the mass is proportional to the square of the flight time of the ion.

The ion trap analyzer and the quadrupole analyzer are based on similar principles, as both use an electric field to separate ions via *m*/*z* ratios and then trap ions in a controlled manner. In ion trap analyzers, the ions are first captured or trapped for a specific interval [180]. A quadrupole ion trap analyzer has a ring electrode and an endcap electrode. The ion trap is operated by a fixed radio frequency supplied to the ring electrode, while the endcap electrode has a constant DC current (usually = 0). An ion trap operates by storing ions in a trap and manipulating them by using DC and RF electric fields in a series of carefully timed events [181]. The main difference between an ion trap and a quadrupole analyzer is that an ion trap is capable of trapping ions for long periods of time (milliseconds to days), providing plenty of time for ions to fall apart spontaneously (unimolecular decomposition) or to experience undesirable interactions with other ions (space charge effects) or with neutral molecules (ion-molecule reactions). This long trapping time provides some unique capabilities, such as extended-length MS/MS experiments and high sensitivity.

In addition to ESI, other soft ionization mass spectrometric methods have been utilized to detect metabolites present in biofluids. Both ESI and matrix-assisted laser desorption ionization (MALDI) are sensitive analytical techniques that use analyte concentrations as low as one picomolar [173]. MALDI differs from ESI by the state in which a sample is introduced to the ion source; ESI uses a solvated sample that is infused into the instrument, whereas the solid state is typically used in MALDI. Therefore, when interfaced with a rapid analytical technique, such as gas or liquid chromatography, ESI is possibly more efficient for quantitative measurements [173].

#### 4.2.2. Gas and Liquid Chromatography Coupled with Mass Spectrometry

To date, HPLC is one of the most popular analytic techniques because it can separate, identify, and quantify compounds that are present in any sample that can be dissolved in a liquid [182]. HPLC uses a liquid mobile phase to transport analytes through a packed stationary phase column. In addition, HPLC is equipped with a detector that is used to respond to a physiochemical property of an analyte [183]. Unfortunately, no single analytical platform can completely identify and quantify all molecules in a sample. The advantages of MS, NMR, and magnetic resonance imaging (MRI) are elucidated in Table 2. In metabolomics, GC-MS is the gold standard for identifying and quantitating small molecules (<650 Da). However, GC-MS is limited to volatile compounds. It has almost 50 years of established protocols for metabolite analyses (e.g., sugars [184], amino acids [185], sterols [186], hormones [187], catecholamines [188], hydroxyl acids [189], fatty acids [188], aromatics [190], and many other intermediates of primary metabolism). Nonetheless, it is biased against nonvolatile, high-molecular-mass metabolites [120].

#### 4.2.3. Targeted vs. Untargeted Metabolomic Assays for Huntington’s Disease

By identifying and characterizing metabolic pathways and biomarkers linked with the progression of HD, we can expand our understanding of the pathophysiology of the disease and develop novel therapies [191]. Targeted and untargeted metabolomic methods are valuable tools for analyzing low-molecular-weight molecules called metabolites in biological samples and can provide a better understanding of disease etiopathophysiology [192,193].

**Figure 6:**
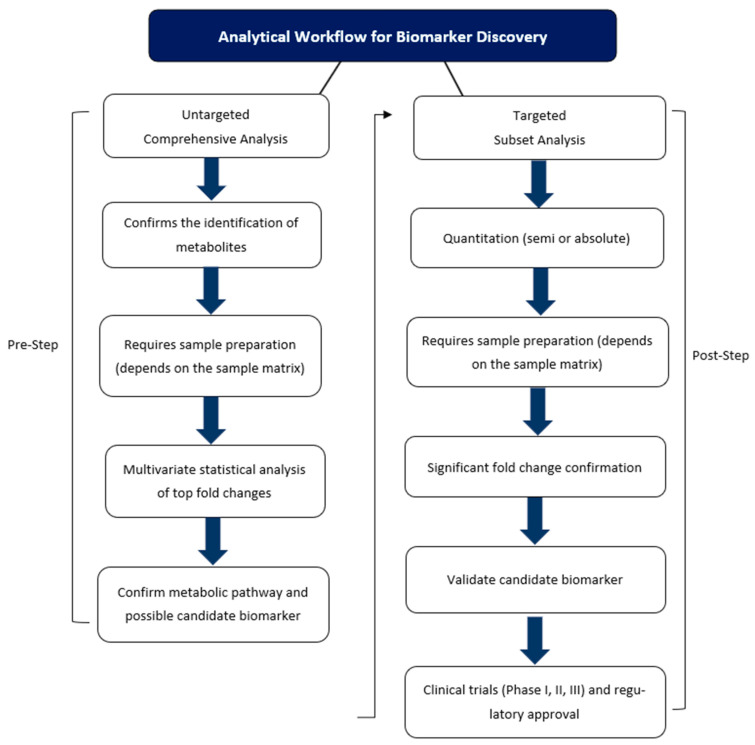
Schematic diagram of how omics platforms are used for potential biomarker discovery. The workflow is based on the LC/MS platform.

As in other omics fields, metabolomic and lipidomic analysis consists of targeted and untargeted approaches. In general, untargeted analysis focuses on profiling (global or fingerprints) the metabolites in a sample, whereas targeted analysis focuses on quantitating selected metabolites (Figure 6). The fundamental aim of global untargeted and targeted metabolomics is now realistic and achievable due to advances in analytical techniques [194,195,196]. The workflow of global and targeted metabolomics is similar. In MS-based metabolomics, generally, there is an extraction step, followed by sample preparation, and analysis by liquid or gas chromatography combined with mass spectrometry. Sample preparation steps for NMR-based metabolomics vary depending on whether solution or solid-state NMR is being utilized. Metabolomics studies using solution-based NMR are usually performed on biofluids and cell or tissue extracts, which require either sample preparation or sample extraction followed by sample preparation [197]. NMR-based metabolomics has a clear advantage for tissue metabolomics in that ^1^H high-resolution magic-angle spinning can be used for direct sample analysis and requires no sample preparation [198].

When considering the general workflow of a metabolomics project, one should keep in mind that at the analytical level, the accurate and complete measurement of the metabolome is a challenging task [199]. Challenges include the diversity of metabolites at the physico-chemical level, the broad range of concentrations, and the associated dynamic range for detection. Moreover, the analysis of complex biological matrices is compromised by the presence of macromolecules, high ionic strength, and sample heterogeneity. Therefore, it is crucial to pretreat the samples to minimize these issues and make the biological samples compatible with the analytical platform. However, the more complex and time-consuming such a treatment is, the higher the risk of altering the sample (in terms of metabolite composition), resulting in a higher risk of experimental variability. Therefore, having a cheap and reproducible sample preparation step requiring minimal effort is an essential step toward producing reliable results independent of the operator and analytical instrument. By implementing such methods, it is possible to reduce unwanted artifacts and variations in the metabolomics dataset.

The final steps of the overall process of metabolomic analysis consist of the correct identification of metabolites followed by data analysis. Untargeted metabolomics is first used to acquire metabolomics data because it provides the widest overview of a given metabolome without requiring a priori knowledge of the sample. However, the type of metabolites measured is directly affected by the extraction method, the chromatographic separation technique (liquid vs. gas chromatography), and the applied mass analyzers, whether triple quadrupole (QQQ), quadrupole combined with time-of-flight, or ion trap. The main challenges associated with untargeted metabolomics are: (i) the inherent difficulties associated with accurate metabolite identification such as low signal-to-noise ratios, unavailability of chemical standards, and a lack of database coverage [200]; (ii) computationally demanding tasks [201], (iii) a variation in data between instruments, and (iv) the standardization of protocols and methods to allow for the integration and comparison of data and results between research groups [202].

Targeted metabolomics measures a defined set of metabolites that are annotated with known chemical characteristics and biochemistry. By using internal standards, analysis can be performed quantitatively or semi-quantitatively. This targeted method utilizes a wide range of metabolic enzymes, their kinetics, and end products, and requires a thorough understanding of known biochemical pathways (247). The use of targeted metabolomics can optimize sample preparation by reducing the disadvantages of having a large number of biochemical metabolites in the assay. Additionally, because the evaluated molecules and species have been distinctly determined, further analyses will not be needed in the next stage. Analyzing the list of analytes mentioned above can uncover connections between metabolites under specific physiological conditions.

When LC/MS is used for targeted metabolomics, several key factors affect the accuracy of the test and resulting data. For example, it is important to choose the correct ionization parameters. Electrospray ionization mass spectrometry (ESI) is one of the most widely used instruments for ionizing small molecules in metabolic research utilizing LC/MS. ESI facilitates the use of MS for identifying nonvolatile, high-quality molecules. The main advantage of ESI is that chemical derivatization is not needed to increase volatility and minimize the fragmentation of analytes, which simplifies the analysis and interpretation of complex mixtures. However, ESI also has some drawbacks—most importantly, ion suppression in the analysis of complex molecular mixtures [203]. During ionization, in analytes with suffering chargeability, ion quenching occurs; hence, the ionization efficiency of a single analyte depends on its chemical properties [204].This problem can be especially complicated because the suppression of ions occurs when impeding compounds are not visible in the MS spectrum. Therefore, the quantification should presume that the mixture among the analyzed sample classes is approximately the same. Thus, when conducting experiments, it is important to compare only specimens from the identical matrix; for example, it would be inappropriate to compare tissue extract samples with plasma samples. The effect of the suppression of ions can be decreased by separating the analyte by one or more chromatographic methods before MS [205].

To assess the contribution of biological variation compared with technical variation, a set of serum/plasma samples is obtained before and after the experiment, and metabolome products are extracted, reconstituted, and then added to an internal standard, which is labeled with an isotope for LC/MS study. At the same time, the variance of LC/MS interpretation and sample arrangements can be evaluated by taking an aliquot of the serum/plasma sample from each sample set and combining them to form a single solution. From this combined solution, a subset can be extracted, recovered, and then run through LC/MS. A second subset of this mixed solution can be extracted, recovered, and reassembled to provide a single sample, which can then be analyzed in duplicate by LC/MS to evaluate the role of LC/MS analysis in the variance overall. At the end of the experiments, the coefficient of variation (CV; 100 × SD/average of the data) of biological variance, sample preparation variance, and variance in LC/MS/MS are calculated [206].

Targeted metabolomics contributes precision and depth to the analysis by focusing on known metabolites associated with the disease. Simultaneously, untargeted metabolomics broadens the investigative scope, enabling the detection of novel biomarkers and providing a holistic understanding of the global metabolic perturbations occurring in Huntington’s disease. By synergistically integrating these approaches, researchers can navigate the complexity of Huntington’s disease progression with a more comprehensive toolkit. This not only enhances the accuracy of quantitative measurements for known metabolites but also opens avenues for the discovery of novel indicators and a more profound comprehension of the intricate metabolic dynamics underlying the disease. This integrative strategy, therefore, proves invaluable in advancing our understanding of Huntington’s disease at both a detailed and holistic level.

**Figure 7:**
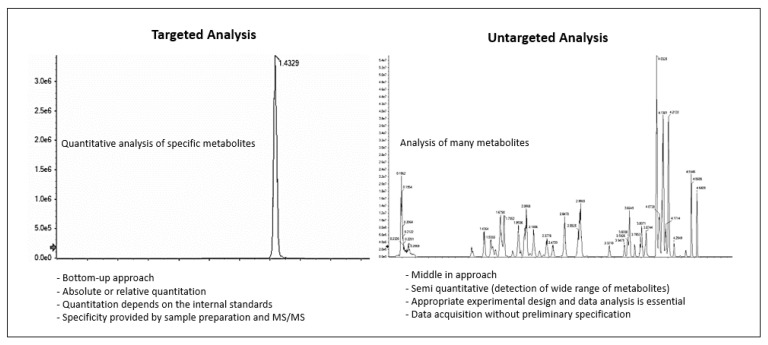
Difference between targeted and untargeted analysis. Untargeted approach employs analysis of all metabolites detected by mass spectrometer, whereas targeted approach only focuses on known metabolites [121,207].

Untargeted analysis is a complete testing of all biochemical analytes measured in the sample, including unknown chemicals (Figure 7). Untargeted metabolomics must be used in conjunction with complex chemometric techniques (multivariate analysis) because of its comprehensive features, to reduce the resulting large data set to a smaller set of managed signals. The signals obtained need to be labeled using silica gel libraries or by comparing them with experimental studies, and then the analytes corresponding to those signals must be identified by analytical chemistry. Targeted analysis provides an opportunity to rediscover targets because the coverage of metabolites is limited only by the natural sensitivity and specificity of the sample preparation method and the analytical method used. Nevertheless, the main disadvantages of untargeted analysis are (i) the protocol and processing time to operate large data, (ii) the difficulty of characterizing and identifying small molecules that are unknown, (iii) the reliance on the specificity and sensitivity of the analytical technique utilized, and (iv) a bias against the detection of high-abundance molecules [206]. Different analytical platforms generate diverse types of data that need specific data processing and analysis workflows. The effectiveness of data interpretation can be influenced by the chosen platform, making it necessary to develop tailored data processing strategies. Targeted metabolomics contributes precision and depth to the analysis by focusing on known metabolites associated with the disease. Simultaneously, untargeted metabolomics broadens the investigative scope, enabling the detection of novel biomarkers and providing a holistic understanding of the global metabolic perturbations occurring in Huntington’s disease. By synergistically integrating these approaches, researchers can navigate the complexity of Huntington’s disease progression with a more comprehensive toolkit. This not only enhances the accuracy of quantitative measurements for known metabolites but also opens avenues for the discovery of novel indicators and a more profound comprehension of the intricate metabolic dynamics underlying the disease. This integrative strategy, therefore, proves invaluable in advancing our understanding of Huntington’s disease at both a detailed and holistic level (Table 3).

In untargeted metabolomics studies, there are two broad methods for data collection. The primary procedure uses MS1 full-scan (injection, ionization, acceleration, and analyses of a sample by mass spectrometry) to create correct mass measurements and/or identify characteristics of individual molecules for statistical calculations, followed by data collection through data-dependent sample subsets (i.e., data-dependent acquisition [DDA]) to guide identification. Similar to traditional proteomics methods, the metabolic DDA method produces a pattern of metabolite fragments with the greatest intensity of signals. The second method for untargeted metabolomics is based on data-independent acquisition (DIA), where the workflow simultaneously exacts mass at high and low collision energies (MS(E)) [208] or in a limited mass range using the sequential window acquisition of theoretical fragment ion spectra mass spectrometry (SWATH) [209], which integrates the complete MS1 and MS/MS cleavage of all precursors. The DIA method generates a complex fragment spectrum, and the relationship between the product and precursor can be difficult to interpret. In subsequent phases of data analysis, the ion fragment must be matched with the pre-ions based on mass, drift time, and retention time. The fragment data provided by DIA have nothing to do with the signal strength of metabolites. Both DIA and DDA methods eventually determine properties through descriptors such as m/z ratio, retention time, and drift time. Finally, in the identification phase of analysis, databases are searched for starting ions and corresponding fragment ions to determine the identity of the metabolites.

One of the main advantages of untargeted metabolomics is that data can be collected without prior knowledge of what the metabolites might be. This comes with a caveat that the sample preparation and analytic methods qualitatively affect the results obtained. Due to the nature of metabolites [210,211], parameters, instrument platforms, separation methods, and sample preparation steps affect the subset of identified metabolites.

The workflow for untargeted metabolomics involves many elucidated phases, including peak detection, retention time alignment, noise filtering, peak deconvolution, and description of function. Principally, the characteristics of substances identified by untargeted analysis do not always correspond to metabolites. Molecules that are similar to metabolites (e.g., adducts, neutral losses, isotopes) can have different *m*/*z* values. In order to reach a biochemical conclusion from untargeted metabolomics, metabolites must be identified. To identify analytes, experimental MS/MS or MS1 data can be searched by using public databases (e.g., METLIN [212], mzCloud (https://www.mzcloud.org, accessed on 1 August 2020), GNPS (http://gnps.ucsd.edu, accessed on 1 August 2020), ChemSpider (http://www.chemspider.com, accessed on 1 August 2020), MassBank [41], Human Metabolome DataBase (HMDB) [213], and LipidBlast [214]) or a fee-based database (e.g., NIST Mass Spectral Library (http://chemdata.nist.gov, accessed on 1 August 2020). Without commercial software, it is often impossible to batch-search the MS/MS fragment mass spectra contained in these databases. Given the large number of libraries it is necessary to search to maximize the coverage of metabolites, bioinformatics is needed to eliminate or reduce redundancy. Because the names of metabolites have not been fully standardized and because of large differences between databases, this process can be complicated. The biochemical understanding and analysis of the data collected for metabolomics and the final systems biology research rely on the ability to correctly define all the metabolites so that they can be located through networks and pathways.

Due to the large amount of data generated, it is difficult to visualize and interpret the data of untargeted metabolomics experiments. Most available analytical tools require that different kinds of recognized metabolites be combined with biochemical/biological knowledge [215,216,217]. Novel technologies based on combining systems biology instruments have been introduced to place small molecules in the biological environment. For instance, metabolic and genomic data are integrated into a mining workflow to recognize promising drug candidates [218]. In contrast, targeted metabolomics is more selective than untargeted metabolomics, and metabolites are analyzed a priori based on information that will develop and optimize methods for analyzing specific metabolites and target metabolic pathways using internal or external reference compounds. By providing analytical confirmation, such an approach can be utilized to determine accurate amounts of biochemical derivatives detected by untargeted metabolomics. Targeted analysis is needed to identify a whole-picture metabolomics system and to verify and extend the outcomes of untargeted analysis [219]. Data obtained by targeted analysis can then be used as input variables for statistical analysis. The core panel of metabolomics contains many metabolic mediators, neuromodulators, organic acids, drug metabolites, carboxylic acids, and lipids. These types of panels are designed to meet the needs of case researchers.

Many substantive studies have been conducted to improve the identification of metabolites [220] and to improve computing solutions [221] and harmonize the protocols and methods [222,223]. From a clinical perspective, each single-board or multi-board test must be based on an absolute quantitative method for appropriate quality control, documentation, and method validation. A standardized commercial kit with a complete calibration curve that returns quantitative measurements can be an attractive complement to more exploratory, untargeted methods. However, serum and plasma are the main samples used by these kits, which are usually developed and validated using traditional QQQ mass spectrometry and have been used for cerebrospinal fluid (CSF) only in a few cases [223,224].

The combination of HRMS LC-MS, NMR and neuroimaging, along with other advanced techniques, allows for multimodal data integration. This holistic approach provides a comprehensive understanding of the molecular, metabolic, and structural changes occurring in Huntington’s disease, offering a more complete picture of the disease mechanisms. These advanced techniques offer a deeper understanding of the molecular and metabolic changes associated with Huntington’s disease. This includes changes in levels of neurotransmitters, molecular and structural information, energy metabolites, and other small molecules crucial for cellular function, enabling researchers to identify biomarkers and dynamics and develop targeted interventions for this complex neurodegenerative disorder. This temporal information is critical for understanding how metabolic pathways are dysregulated over time and may provide insights into disease stages and potential intervention points [55,76,172]. NMR and imaging offer non-invasive structural and functional insights into the brain, allowing researchers to observe changes in neuronal structures and functions associated with Huntington’s disease. This includes the assessment of brain atrophy, connectivity alterations, and changes in neurotransmitter levels. Neuroimaging enables the in vivo monitoring of specific biomarkers associated with Huntington’s disease. This allows for the real-time observation of disease progression and the effects of potential therapeutic interventions, providing a dynamic view of the disease at the molecular level. 

## 5. Conclusions

The study of Huntington’s disease (HD) through the lens of metabolomics has emerged as a critical avenue for unraveling the intricacies of this neurodegenerative disorder. Despite the discovery of the HD mutation in 1993, the absence of disease-modifying treatments underscores the urgency in comprehending the molecular and cellular underpinnings of HD. Metabolomics, with its distinctive advantages, has played a pivotal role in advancing our understanding of the disease. The precision and sensitivity offered by targeted metabolomics have allowed for in-depth analyses of specific metabolites associated with HD. This focused approach not only aids in the identification of biomarkers but also contributes to our understanding of well-established metabolic pathways affected by the disease. On the other hand, untargeted metabolomics, with its capacity for comprehensive and unbiased exploration, has facilitated the discovery of novel biomarkers and provided a holistic view of the global metabolic alterations in HD. In the period from 2001 to 2023, many studies have signified a crucial timeline where metabolomic investigations have thrived, contributing valuable insights into HD. These insights, ranging from changes in the metabolome associated with HD to the identification of potential non-invasive biomarkers, hold significant promise for the development of diagnostic tools and prognosis assessments, as well as the monitoring of therapeutic efficacy. As we navigate the complex landscape of Huntington’s disease, metabolomics stands as a beacon, offering a nuanced understanding of the molecular signatures and metabolic shifts that define this condition. It is through the integration of targeted and untargeted metabolomics approaches that we can harness the full spectrum of analytical capabilities, ultimately paving the way for the development of novel treatments and personalized therapeutic strategies for individuals affected by HD.

## Figures and Tables

**Table 1:** Types of NMR that can be used for lipidomic and metabolomic studies, by incorporating magnets of varying strengths for analysis.

Frequency (MHz)	Findings	Reference
400	A ^1^H-NMR-based metabolomics approach was used in a temporal region–specific investigation of the metabolome of neuron-specific 26S proteasome knockout mice characterized by progressive neurodegeneration and Lewy body-like inclusions in the forebrain.	[133]
500	^1^H-NMR metabolic profiling was used to characterize metabolic aberrations in a yeast model of HD that is attributable to the mutant huntingtin protein’s gain-of-toxic-function effects.	[134]
600	^1^H-NMR metabolic profiling of postmortem striatum and frontal lobe from HD patients provided new insights into disease pathophysiology.	[87]
700	Proton NMR spectroscopic investigation of serum and cerebrospinal fluid (CSF) taken from pre-symptomatic HD transgenic rats and their wildtype littermates suggested a defect in energy metabolism.	[67]
800	^1^H-NMR spectroscopic analyses of CSF specimens were conducted to develop a biomarker panel for multiple sclerosis (MS); it yielded reproducible detection of 15 metabolites from MS (n = 15) and non-MS (n = 17) patients.	[67,108]

**Table 2:** Summary of the analytical technique that is used in relation to Huntington’s disease.

Technique	Mass Spectrometry (MS)	Nuclear Magnetic Resonance (NMR)	Magnetic Resonance Imaging (MRI)
**Fields used in**	Diverse use (proteomics, metabolomics, lipidomics, drug discovery, toxicology)	Diverse use (proteomics, metabolomics, lipidomics, drug discovery, toxicology)	Radiation oncology, neurologic disease
**Detection mechanism**	Chemical compounds are converted into gas phase molecules, and their mass-to-charge (*m*/*z*) ratio is measured.	Electromagnetic radiation sources can be tuned to different frequencies; therefore, NMR acquires spectra from different kinds of nuclei.	MRI uses natural magnetic properties and radio waves to generate images of the organs of the body. A single proton in a hydrogen nucleus is utilize due to its abundance in water and fat.
**Compatible**	Solid/Gas/Liquid	Solid/Liquid	Solid
**Sensitivity**	High (nanogram to picogram)	Low (milligram to nanogram)	90.5% sensitive
**Selectivity**	Both targeted (selective and non-targeted (non-selective) assays can be performed.	Non-selective analysis	Selective
**Reproducibility**	Moderate to high, depending on the sample clean up, per analyte of interest biochemical properties	High	Reproducible
**Sample preparation**	Time-consuming and depends on the sample matrix. Liquid/liquid/ Solid phase extraction or chemical derivatization can be used.	Compared to MS, NMR sample preparation is minimal.	N/A
**Sample volume**	**Biological fluid:** 5–500 µL (depends on the assay) **Cells:** 3–10 million **Tissue:** 10–25 mg	**Biological fluid:** 50–500 µL (depends on the assay) **Cells:** 15–25 million **Tissue:** 25 mg to check	Physical presence of the patients
**Sample Matrix**	Tissue, Cells, Serum, Saliva, Tears, Hair, Ear Wax, CSF, Plasma, Urine, Whole Blood	Tissue, Cells, Serum, Saliva, Tears, Hair, Ear Wax, CSF, Plasma, Urine, Whole Blood	Organ imaging
**Identification**	100 to more than 1000 in a single experiment	40–200 depending on spectral resolution	Target to certain metabolites
**Quantitation**	Qualitative and quantitative analysis can be performed. Needs isotope-labeled standards and calibration curves for each analyte for absolute quantitation	Absolute quantitation; however, requires a standard of known concentration	N/A
**Advantages**	**GC-MS:** Relatively inexpensive, modest sample size, great sensitivity, a large body of software available and databases for metabolite ID. **LC-MS:** Detects most organic and inorganic molecules, minimal sample size required, direct injection can be possible, has the potential to detect largest portion of the metabolome and lipidome	Quantitative (^1^H NMR), non-destructive, fast, requires no derivatization, detects all organic classes, allows ID of novel metabolites, robust, large body of software and database available for metabolite ID	MRI Scan provide detailed images of soft tissues, organs and bones allowing for better visualization and diagnosis of various medical conditions. It’s a non-invasive procedure and do not require any surgical procedures.
**Disadvantages**	**GC-MS:** time-consuming, novel ID is difficult, longer run time. **LC-MS:** time-consuming and longer run time, as it depends on the type of LC used.	Less sensitive than MS and expensive to maintain	MRI scan is time consuming and inconvenient for patients who may need to lie still during the procedure.

**Table 3:** The advantages and disadvantages of targeted and untargeted metabolomics in relation to biological disease.

Advantage/Disadvantage	Targeted	Untargeted
Advantage	High Sensitivity and Specificity	Comprehensive Analysis
	Quantitative Accuracy	Discovery of Novel Biomarkers
	Data Interpretation is Easier	Systems Biology Approach
Disadvantage	Limited Coverage	Data Complexity and Quantitative Challenges
	Less Comprehensive	Lower Sensitivity
